# Enantiomeric Effect of d-Amino Acid Substitution on the Mechanism of Action of α-Helical Membrane-Active Peptides

**DOI:** 10.3390/ijms19010067

**Published:** 2017-12-27

**Authors:** Shiyu Sun, Guangxu Zhao, Yibing Huang, Mingjun Cai, Qiuyan Yan, Hongda Wang, Yuxin Chen

**Affiliations:** 1 Key Laboratory for Molecular Enzymology and Engineering of the Ministry of Education, Jilin University, Changchun 130012, China; sunshiyu1988@126.com (S.S.); 15643620230@163.com (G.Z.); huangyibing@jlu.edu.cn (Y.H.); 2 School of Life Sciences, Jilin University, Changchun 130012, China; 3 National Engineering Laboratory for AIDS Vaccine, Jilin University, Changchun 130012, China; 4 State Key Laboratory of Electroanalytical Chemistry, Changchun Institute of Applied Chemistry, Chinese Academy of Sciences, Changchun 130022, Jilin, China; caimingjun@ciac.ac.cn (M.C.); qyyan@ciac.ac.cn (Q.Y.); 5 University of Chinese Academy of Sciences, Beijing 100049, China

**Keywords:** atomic force microscopy, membrane-active peptide, helicity, hydrophobicity

## Abstract

V13K, a 26-residue peptide, has been shown to have strong antimicrobial activity, negligible hemolytic activity, and significant anticancer activity. In the present work, V13K was used as the framework to investigate the influence of helicity, as influenced by d-amino acid substitutions in the center of the peptide polar and non-polar faces of the amphipathic helix, on biological activity. The antibacterial and anticancer activities of the peptides were investigated. Atomic force microscopy and other biophysical methods were used to investigate the effect of peptide helicity on biological activity. The results showed the importance of suitable and rational modification of membrane-active peptides, based on helicity, in optimizing potential biological activity.

## 1. Introduction

Despite recent progress in the treatment of bacterial infections and tumors, the development of resistance and the non-specific toxicity of many conventional drugs still preclude their clinical application. Therefore, developing new therapeutic agents that render cells non-resistant to treatments for bacterial infection and/or cancer will propel the development of more effective antimicrobial and anticancer therapeutics [1,2,3]. Cationic membrane-active peptides (MAPs) have attracted recent attention mainly due to their novel mode of action, which disrupts the cytoplasmic membrane via micellization or pore formation [4]. As the cytoplasmic membrane is the main target of most cationic peptides, it is not surprising that bacterial resistance could not easily develop on MAPs.

A number of factors including hydrophobicity, net charge, amphipathicity, the nature of the secondary structure in the membrane, and oligomerization ability have been proven to be important for the biological activity of MAPs [5,6,7]. In a previous study, the hydrophobicity and net charge were demonstrated to dramatically influence the biological activities of peptides [8]. Papo et al. showed that peptides with d-amino acid substitutions instead of l-amino acids could significantly increase antibacterial activity and reduce the cytotoxic effects on mammalian cells [9,10]. Based on structure/activity studies from a previous study [11], we successfully designed a peptide V13K with strong antimicrobial activity and specificity against Gram-positive and Gram-negative bacteria. In this study, V13K was used as the framework to investigate the influence of helicity on biological activity. The helicity was altered by d-amino acid substitutions in the center of the polar and nonpolar faces of the amphipathic helix. The objectives of this study were threefold: first, to test whether the modulation of V13K with d-amino acid substitutions on the polar and non-polar faces alters the biophysical properties, such as helicity; second, to investigate whether these substitutions can further improve the biological activity; and third, to use atomic force microscopy (AFM) to study the interaction of the peptides with bacterial and cancer cells to reveal the mechanism of action of MAPs with different types of cell membranes.

## 2. Results

### 2.1. Peptide Design

**Figure 1:**
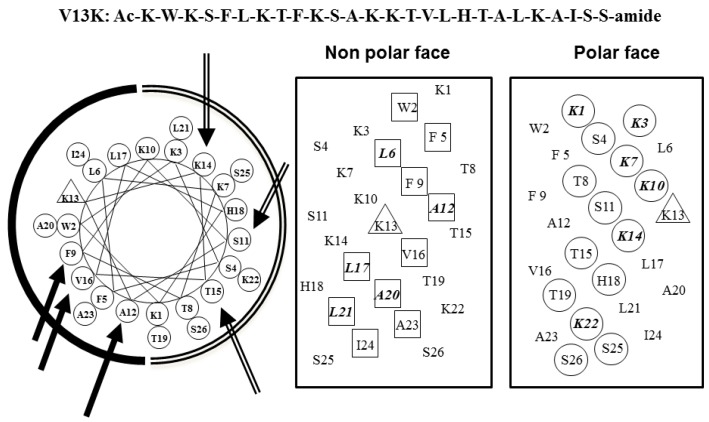
Representation of the parent peptide V13K as helical nets showing the polar/hydrophilic face (circled residues) and non-polar/hydrophobic face (boxed residues); and helical wheel, the lysine residue at position 13 of the sequence is denoted by a triangle. In the helical wheel, the three single substitution sites are shown with solid arrows on the non-polar face (solid arc), and hollow arrows on the polar face (open arc), respectively, One-letter codes are used for the amino acid residues.

V13K is a 26-residue, α-helical, amphipathic, membrane-active peptide with antimicrobial and anticancer activities [11,12]. The peptide sequence, helical net, and helical wheel of V13K are shown in Figure 1. Our previous studies have indicated that peptide hydrophobicity plays a critical role in the antimicrobial activity of peptides [11,13], and amino acid substitutions in central locations of V13K can affect the antimicrobial and hemolytic activity of peptides [11,14]. Huang et al. demonstrated that d-peptides exhibited stronger antimicrobial activity than the corresponding l-enantiomers [15]. In the present study, we substituted d-amino acids at sites near the center of the hydrophobic/hydrophilic face of the helix to investigate the effect of d-amino acid substitutions on the secondary structure of the peptides. Centrally-located substitutions have been shown to have a marked effect on the secondary structure of peptides [16,17]. The peptide analogs K14_D_, S11_D_/K14_D_, K14_D_/T15_D_, and S11_D_/K14_D_/T15_D_ were obtained from d-amino acids substitutions at the 11, 14, and 15 positions on the polar face. The peptide analogs A12_D_, F9_D_/A12_D_, A12_D_/V16_D_, and F9_D_/A12_D_/V16_D_ were obtained from d-amino acids substitutions at the nine, 12, and 16 positions on the non-polar face. The sequences of the peptide analogs are shown in Table 1.

### 2.2. Peptide Secondary Structure

The influence of the amino acid substitutions on the peptide secondary structure were investigated using circular dichroism (CD) spectroscopy under benign conditions (KP buffer) and under conditions designed to mimic the hydrophobic environment of the membrane (KP buffer with 50% TFE). d-amino acid substitutions reduced the helicity of the peptide analogs relative to that of peptide V13K, in both KP buffer and KP buffer with 50% TFE. Moreover, increasing the number of d-amino acid substitutions, on either the polar or non-polar face, resulted in a corresponding gradual decrease in helicity. As shown in Table 2 and Figure S1, the values of the relative helicity of peptides with polar face substitutions are in the order of K14_D_ (76.99%) > S11_D_/K14_D_ (51.33%) > S11_D_/K14_D_/T15_D_ (38.67%). Similarly, the relative helicity of the peptides with substitutions on the non-polar face follows the same trend: A12_D_ (70.83%) > F9_D_/A12_D_ (38.26%) > F9_D_/A12_D_/V16_D_ (36.41%). Since the values of the relative helicity of the peptides with d-amino acid substitutions on the non-polar face were less than those peptide analogs with the same number of d-amino acid substitutions on the polar face, it is clear that the amino acid residues on the non-polar face may be more important for stabilizing the α-helical structure than residues on the polar face. In addition, the relative helicity values of peptides S11_D_/K14_D_ (51.33%) and F9_D_/A12_D_ (38.26%) were lower than those of the peptides K14_D_/T15_D_ (56.51%) and A12_D_/V16_D_ (46.42%), respectively, suggesting that N-terminal amino acids may play an important role in maintaining the α-helical structure of V13K.

### 2.3. Peptide Hydrophobicity

The RP-HPLC retention time has been widely used to represent the relative hydrophobicity of peptides in many studies [11,13,18,19].The relative hydrophobicity of the peptides was determined by the RP-HPLC retention times at 25 °C. The change in hydrophobicity of the peptides caused by d-amino acid substitutions was mainly due to the disruption of the helical structure. As shown in Table 2, the RP-HPLC retention time (*t_R_*) correlated with the number of d-amino acid substitutions on both the polar and non-polar faces, that is, the more d-amino acid substitutions the less hydrophobic the peptide.

### 2.4. Biological Activity

The influence of d-amino acid substitutions on the biological activity of the peptides was examined. In this study, the peptide analogs exhibited reduced biological activity, including antimicrobial activity (as indicated by MIC values) and anticancer activity (as indicated by IC_50_ values) compared with the parent peptide V13K (Table 3 and Table 4). Interestingly, increasing the number of d-amino acid substitutions produced a trend of an increase in the values of the MIC and IC_50_ values, respectively, indicating a decrease in antimicrobial and anticancer activity. Moreover, the same number of amino acid substitutions on the polar face of the parent peptide resulted in less of a decrease in biological activity than amino acid substitutions on the non-polar face, indicating the importance of the non-polar face of the peptide for biological activity. Cellular toxicity was assessed by the values for the hemolysis of human red blood cells. The peptide analogs showed negligible changes in hemolysis compared with V13K, except for A12_D_/V16_D_ and F9_D_/A12_D_/V16_D_, which exhibited much less hemolytic activity than V13K (Table 3).

### 2.5. Cell Membrane Permeabilization

**Figure 2:**
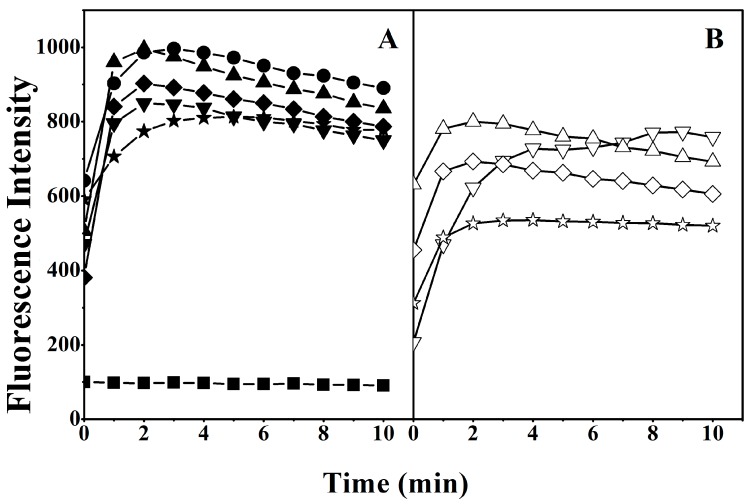
Membrane permeabilization assay of the peptides. Outer membrane permeabilization induced by the peptides was detected by NPN uptake in *E. coli*. Panel (**A**) denotes the peptides with substitutions on the polar face and Panel (**B**) denotes the peptides with substitutions on the non-polar face. Symbols used are as follows: ● for V13K; ▲ for K14_D_; ▼ for S11_D_/K14_D_; ◆ for K14_D_/T15_D_; ★ for S11_D_/T14_D_/T15_D_; △ for A12_D_; ∇ for F9_D_/A12_D_; ◊ for A12_D_/V16_D_; and ☆ for F9_D_/A12_D_/V16_D_; ■ for Control.

To explore the potential role of the helicity of the peptides in damaging cell membranes, we investigated the disruption of the outer membrane of *Escherichia coli* (*E. coli*) by quantifying the fluorescence intensity of 1-*N*-phenylnaphthylamine (NPN), which is a hydrophobic fluorescent probe. The intensity of NPN is weak in a hydrophilic environment. The fluorescence intensity of NPN gradually increases as it is exposed to a more hydrophobic environment due to damage to the bacterial outer membrane by MAPs [20]. As shown in Figure 2, the fluorescence intensity of NPN was enhanced in the outer membranes treated with peptides with high helicity, such as V13K and K14_D_, suggesting that peptides with high helicity disrupted the biomembrane more than those with less helicity.

**Figure 3:**
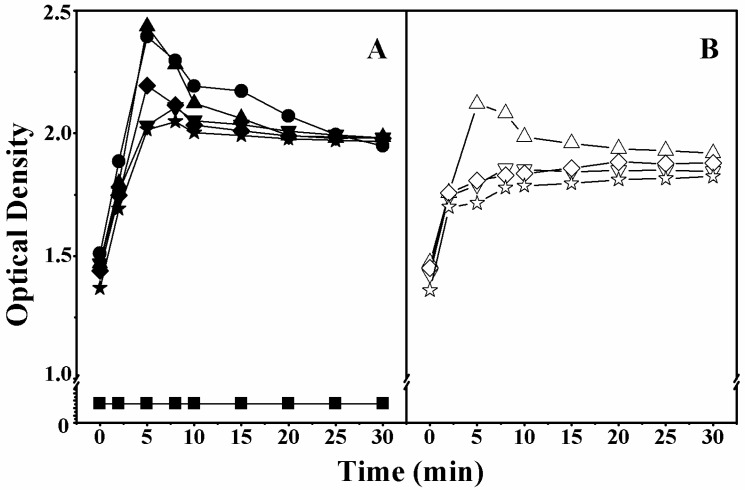
The effect of peptides on the inner membrane permeabilization of *E. coli* ML-35. Cytoplasmic β-galactosidase activity (measured by the absorbance at OD_420nm_) from *E. coli* ML-35 treated with peptides. Panel (**A**) denotes the peptides with substitutions on the polar face and Panel (**B**) denotes the peptides with substitutions on the non-polar face. Symbols used are as follows: ● for V13K;▲ for K14_D_; ▼ for S11_D_/K14_D_; ◆ for K14_D_/T15_D_; ★ for S11_D_/T14_D_/T15_D_; △ for A12_D_; ∇ for F9_D_/A12_D_; ◊ for A12_D_/V16_D_; and ☆ for F9_D_/A12_D_/V16_D_; ■ for Control.

The peptide-induced disruption of the inner membrane of the lactose permease deficient bacteria *E. coli* ML-35 was also investigated. When the inner cell membrane is damaged, the lactose analog ο-nitrophenyl β-d-galactopyranoside (ONPG) rapidly enters into the cells and is hydrolyzed into galactose and o-nitrophenol by β-galactosidase, giving a yellow color. Thus, we can evaluate the disruption of inner bacterial membranes by monitoring the fluorescence of o-nitrophenol at an absorbance of OD_420nm_ [21]. As shown in Figure 3, compared to peptides with multiple d-amino acid substitutions, peptides with single d-amino acid substitutions (on both the polar and non-polar faces) exhibited higher fluorescence intensity. These findings further confirmed the importance of peptide helicity in the disruption of bacterial membranes.

### 2.6. Interaction of Peptides with Liposome Model Membranes

To investigate the interactions between MAPs and different types of cell membranes, we established three different types of LUVs (Large Unilamellar Vesicles) with PC/PG (7:3, *w*/*w*), PC/Chol (8:1, *w*/*w*), and PC/SM/PE/PS/Chol (4.35:4.35:1:0.3:1, *w*/*w*) to mimic anionic prokaryotic membranes, zwitterionic eukaryotic membranes, and cancer cell membranes, respectively [8]. Tryptophan in peptide sequences is a well-characterized probe to detect the interactions of peptides and LUVs as model membranes. The fluorescence intensity increases and the fluorescence emission maxima of tryptophan shifts to the side of blue light when a peptide containing tryptophan enters into the hydrophobic environment of the membrane [22]. As shown in Figures S2 and S3 and Table 5, V13K showed a larger blue shift and larger increase in fluorescence intensity in all the LUVs, suggesting that V13K has the strongest interaction with membranes, compared with the analogs with d-amino acid substitutions. In addition, peptides with more d-amino acid substitutions showed smaller blue shifts and lower fluorescence intensities than the peptides with less d-amino acid substitutions.

We further studied the fluorescence intensity of tryptophan in the peptides, and a water soluble potassium iodide (KI) quencher was used to quench the tryptophan fluorescence of peptides in the solution since tryptophan fluorescence would not be quenched when the peptides entered into the hydrophobic LUVs [23]. As shown in Figure S3, the degree of KI quenching of the peptides with high helicity (K14_D_ and A12_D_) is weaker than for peptides with low helicity (S11_D_/K14_D_/T15_D_ and F9_D_/A12_D_/V16_D_), mainly because the peptides with high helicity inserted deeply in the membrane. However, peptides with more d-amino acid substitutions exhibited lower helicity and had difficulty entering into the hydrophobic core of the LUV’s membrane. These findings further confirmed the important role of the helicity of the peptide in cell membrane damage.

### 2.7. AFM Imaging

**Figure 4:**
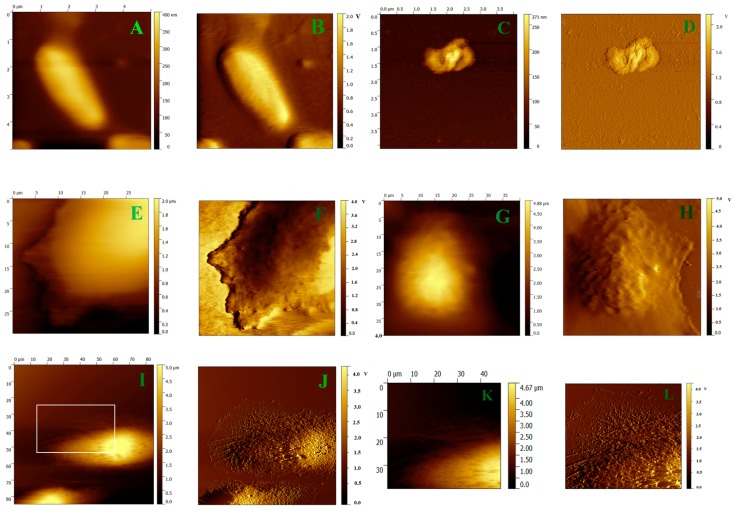
Representative AFM topographic and amplitude images of *E. coli* and HeLa cell membranes before, and after, interaction with the parent peptide V13K. Panel (**A**) shows a topographic image of *E. coli*; Panel (**B**) shows the amplitude image corresponding to Panel (**A**); Panel (**C**) shows a topographic image of *E. coli* interacting with the peptide V13K; Panel (**D**) shows the amplitude image corresponding to Panel (**C**); Panel (**E**) shows a topographic image of an unfixed HeLa cell; Panel (**F**) shows the amplitude image corresponding to Panel (**E**); Panel (**G**) shows a topographic image of a HeLa cell fixed by 4% paraformaldehyde; Panel (**H**) shows the amplitude image corresponding to Panel (**G**); Panel (**I**) shows a topographic image of HeLa interacting with the peptide V13K; Panel (**J**) shows the amplitude image corresponding to Panel (**I**); Panel (**K**) shows magnification of the membrane in Figure 4I; Panel (**L**) shows the amplitude image corresponding to Panel (**K**).

The mechanism of the interaction of the peptides with the different kinds of membranes was further analyzed by AFM. *E. coli* and HeLa cells were imaged to observe the morphologic change of cell membranes with, and without, treatment with V13K. As shown in Figure 4, the topographic and amplitude images of untreated *E. coli* cells exhibited a smooth surface, whereas the cells treated with V13K displayed obvious damage to the morphology of bacterial membrane (Figure 4A–D). Similarly, the surface of untreated cells fixed with, or without, 4% paraformaldehyde were intact and the membrane surface appeared quite smooth (Figure 4E–H). In contrast, pores and cavities were visible on the surface of the cells after treatment with V13K (Figure 4I,J). Furthermore, the magnified image of cells treated with V13K displayed a severely disrupted cell membrane with loss of microvilli and membrane integrity, and exposure of the cytoskeleton (Figure 4K,L).

## 3. Discussion

V13K, obtained by the insertion of a hydrophilic lysine at the center of the non-polar face of the peptide to reduce its hydrophobicity and helicity, is an amphipathic α-helical peptide with strong antimicrobial, negligible hemolytic, and significant anticancer activity [11,12]. In this study, V13K was used as a framework to study the effect of peptide helicity on the mechanism of action of MAPs, and to investigate whether modulating the helicity could further improve the biological activity. Surprisingly, d-amino acid substitutions on either the polar or non-polar face reduced the helicity of V13K peptide analogs resulting in a decrease in biological activity. The hydrophobicity of the analogs was also reduced by the d-amino acid substitutions, compared with peptide V13K. Moreover, the peptide analogs with more d-amino acid substitutions showed weaker helicity and less hydrophobicity. These phenomena are mainly due to the ability of the d-amino acids to disrupt the integrity of the α-helical structure [13].

In this study, peptides with higher helicity showed stronger antimicrobial and anticancer activity (Table 3 and Table 4), which is consistent with previous studies [12,13]. To further explain why higher helicity is associated with greater biological activity, the integrity of outer and inner membranes treated with MAPs was investigated. Membrane permeabilization experiments indicated that the presence of helicity was a critical factor for a peptide to possess promising biological activity (Figure 2 and Figure 3). In addition, the fluorescence intensity and fluorescence emission maxima of tryptophan showed an increasing trend in peptides with greater helicity, in model membranes mimicking anionic prokaryotic, zwitterionic eukaryotic, and cancer cell membranes (Table 5, Figures S2 and S3). These results indicate that the peptides specifically targeted on the prokaryotic and cancer membranes, rather than normal eukaryotic membranes in the model membranes. Moreover, peptides with more d-amino acid substitutions are weaker in helicity and biological activity than the parent peptide V13K, indicating the importance of a suitable modification of MAPs based on helicity in optimizing biological activity. AFM imaging experiments further explored the mechanism of action of the interaction of the membrane-active peptide V13K with prokaryotic and cancer cell membranes (Figure 4). V13K dramatically disrupted both bacterial and tumor cells as evidenced by membrane lysis.

## 4. Materials and Methods 

### 4.1. Reagents

All N-α-Fmoc-protected amino acids, rink amide 4-methylbenzhydrylamine resin, coupling reagents for peptide synthesis, and trifluoroacetic acid (TFA) were purchased from GL Biochem (Shanghai, China). The test strains *Escherichia. coli* (*E. coli*) ATCC25922, *Pseudomonas aeruginosa* (*P. aeruginosa*) ATCC27853, *Staphylococcus aureus* (*S. aureus*) ATCC25923, *Staphylococcus epidermidis* (*S. epidermidis*) ATCC12228, and *E. coli* ML-35 ATCC43827 were purchased from the American Type Culture Collection (Manassas, VA, USA). Red blood cells (RBCs) used in the experiments were extracted from healthy blood donors. Human cervix carcinoma cells (HeLa), human pancreatic carcinoma cell line MIAPaCa-2, HPAC, and BxPC-3 were obtained from the American Type Culture Collection (Manassas, VA, USA). Cholesterol (Chol), chicken egg phosphatidylcholine (Egg PC), porcine brain phosphatidylserine (Brain PS), porcine brain phosphatidylethanolamine (Brain PE), *E. coli* phosphatidylglycerol (*E. coli* PG), and porcine brain sphingomyelin (Brain SM) were purchased from Avanti Polar Lipids, Inc. (Alabaster, AL, USA). Trifluoroethanol (TFE), 1-*N*-phenylnaphthylamine (NPN), and o-nitrophenyl-β-d-galactoside (ONPG) were purchased from Sigma (Beijing, China). 4-(2-hydroxyethyl)-1-piperazineethanesulfonic acid (HEPES) and KI were purchased from Beijing Chemical Works (Beijing, China).

### 4.2. Peptide Synthesis and Purification

All peptides were synthesized by solid-phase peptide synthesis using Fmoc (9-fluorenyl-methoxycarbonyl) chemistry and rink amide 4-methylbenzhydrylamine resin (MBHA resin; 0.8 mmol/g) and purified (>95% purity) by RP-HPLC as described previously [13,24]. Further characterization was performed by mass spectrometry and amino acid analysis.

### 4.3. Circular Dichroism (CD) Analysis

The secondary structures of the peptides were examined using a Jasco J-810 CD spectrometer (Jasco, Easton, MD, USA) at 25 °C as described previously [22]. Briefly, a concentration of 75 μM of peptide was measured in benign buffer (pH 7.0, 100 mM KCl, 50 mM KH_2_PO_4_/K_2_HPO_4_) to mimic a hydrophilic environment, and 50% 2,2,2-trifluoroethanol (TFE) (benign buffer: TFE = 1:1, *vol*/*vol*) to simulate a hydrophobic environment. The mean residue molar ellipticities were calculated according to the equation [θ] = θ/10*l*C_M_n, in which, θ represents the ellipticity in millidegrees, *l* is the optical path length of the cuvette in centimeters, C_M_ is the peptide concentration in mol/L, and n is the number of residues in the peptide. The mean residue molar ellipticities at 222 nm, [θ]_222_ (degree·cm^2^·dmol^−1^), were used to calculate the relative helical content of the peptides.

### 4.4. Antimicrobial Activity Assay

The antimicrobial activity of the peptides was determined by the standard microtiter dilution method and indicated as MIC (minimal inhibitory concentration) as described previously [15]. Briefly, bacteria were cultured in Mueller–Hinton (MH) medium overnight at 37 °C, diluted to a final inoculum of 5 × 10^5^ colony-forming units (CFU)/mL with MH and loaded into 96-well microtiter plates (90 μL/well) with serial dilution of peptides (10 μL/well), with a further incubation at 37 °C for 24 h. MIC was expressed as the lowest peptide concentration that inhibited the growth of bacteria. The antimicrobial assays were repeated in triplicate.

### 4.5. Cell Culture and Analysis

HeLa, MIA PaCa-2, HPAC, and Bxpc-3 cells were maintained in Dulbecco’s modified Eagle medium) with 10% fetal bovine serum, penicillin (100 U/mL), and streptomycin (100 U/mL) in a humid atmosphere at 37 °C with 5% CO_2_. The effect of the peptides on the survival of HeLa, MIA PaCa-2, HPAC, and Bxpc-3 cells was determined by 3-(4,5-dimethyl-2-thiazolyl)-2,5-diphenyl-2H-tetrazolium bromide (MTT) assay, as described in a previous study [25]. Briefly, cells were seeded in 96-well plates at a density of 5000 per well. After overnight incubation, cells were incubated, in the absence, or presence of various concentrations of peptides for 24 h. Then, the formazan crystals formed by MTT were dissolved with dimethyl sulfoxide (DMSO) and the absorbance was determined at 492 nm with a microplate reader (GF-M3000; Gaomi Caihong Analytical Instruments Co., Shandong, China). The results were expressed as IC_50_ values, representing the concentration at which cell viability was reduced by 50%. The cytotoxicity assays were repeated in triplicate.

### 4.6. Hemolytic Activity Assay

The cancer-selective toxicity of the peptides was measured by a hemolytic activity assay as described previously [18,25]. Briefly, healthy human RBCs were collected using an anticoagulation tube with EDTAK2 and centrifuged at 1000 rpm for 5 min. The erythrocytes were washed three times, then diluted to a concentration of 2% in phosphate-buffered saline (PBS). The peptides were serially diluted in PBS and 70 μL of the peptide solutions and 2% RBC suspension were added simultaneously to each well of a 96-well plate (round bottom) for a further incubation at 37 °C for 2 h. Then, the plates were centrifuged for 10 min at 3000 rpm and the supernatant (90 μL) was transferred to a 96-well plate (flat bottom). The absorbance of the supernatant was measured on a microplate reader at 578 nm. Erythrocytes in PBS and distilled water were used as the negative (0%) and positive (100%) controls, respectively. The hemolytic activity was calculated as the percentage of experimental group over positive control (100%), after subtraction of negative control (0%). The hemolytic assays were repeated in triplicate.

### 4.7. Outer Membrane Permeability Assay

The outer membrane permeability of the peptides against *E. coli ATCC25922* was evaluated using the hydrophobic fluorescent probe NPN, as performed previously [20]. In brief, *E. coli* was cultured in LB medium at 37 °C until the absorbance of the bacterial suspension at 600 nm was 0.4–0.6, the bacterial pellet was collected by centrifugation and resuspended in a reaction buffer (pH 7.4, 5 mM HEPES, 5 mM NaN_3_, 0.25 mM NPN) until the absorbance at 600 nm was 0.5. Then, an aliquot of the peptide sample and the bacterial suspension were co-incubated to give a final peptide concentration of 16 μM. The same volume of reaction buffer was used as the negative control. A Shimadzu RF-5301 PC fluorescent spectrometer (excitation wavelength of 350 nm, emission wavelength of 420 nm) was used to collect data continuously for 10 min.

### 4.8. Inner Membrane Permeability Assay

A Bio-Tek Synergy 2 microplate reader (BioTek, Winooski, VT, USA) was used to measure the inner membrane permeabilization of peptides against *E. coli* ML-35. Bacterial cells cultured in LB medium containing 5% lactose were collected and resuspended in sterile water until the absorbance at 420 nm was 1.2. Bacterial suspension (100 μL) was mixed with 10 μL of 30 mM ONPG and peptide to give a final concentration of 32 μM of peptide. A 0.5% NaCl solution was used as the negative control. The absorbance at 420 nm was measured at different time points [21].

### 4.9. Preparation of Large Unilamellar Vesicles (LUVs)

LUVs prepared with PC/PG (7:3 *w*/*w*), PC/Chol (8:1 *w*/*w*), and PC/SM/PE/PS/Chol (4.35:4.35:1:0.3:1 *w*/*w*) were used to mimic prokaryotic cell, normal eukaryotic cell, and cancer cell membranes, respectively. The phospholipids were dissolved in chloroform, dried by nitrogen gas (N_2_), and placed under vacuum overnight to remove organic solvent residues. The lipids were resuspended in 10 mM HEPES and 150 mM NaCl buffer (pH 7.4) by vortex shaking above the phase-transition temperature of the phospholipids, freeze-thawed five times, then passed approximately 20 times through two polycarbonate membranes (0.1 μm) with a mini-extruder above the phase-transition temperature of the phospholipids, until the solutions became transparent. The lipid concentrations were determined by phosphorus analysis [8,22].

### 4.10. Tryptophan Fluorescence and Quenching Assay

The LUVs were prepared and incubated in HEPES buffer (pH 7.4, 10 mM HEPES, 150 mM NaCl) and the molar concentration was adjusted to 100 μM. Each peptide (16 μM) was added to 1 mL of HEPES buffer (pH 7.4) containing 0.1 mM LUV liposomes and the peptide/liposome mixture was allowed to interact at 25 °C for 10 min. A Shimadzu RF-5301PC, (excitation wavelength of 280 nm, emission wavelength of 300–450 nm) was used to measure the tryptophan fluorescence. KI as a quencher was added to the reaction system for a final peptide concentration of 0.02–0.08 M. The experimental data were plotted according to the Stern–Volmer equation: F_0_/F = 1 + Ksv[Q], where F_0_ and F are the fluorescence intensities in the absence, or presence, of a quencher at concentration [Q], respectively, and Ksv is the Stern–Volmer quenching constant [13].

### 4.11. AFM Imaging

The AFM imaging of *E. coli ATCC25922* and HeLa cells before, and after, incubation with V13K were performed using an AFM 5500 (Agilent Technologies, Chandler, AZ, USA). The AFM imaging of *E. coli* was used on the contact mode and the cantilever was used as the B-tip (DNP, B-tip, Bruker AFM probes, California, CA, USA). The AFM imaging of HeLa was used on the AAC mode and the cantilever was used as the D-tip (DNP, D-tip, Bruker AFM probes, Camarillo, California, CA, USA). A single bacterial colony was picked and cultured in Mueller–Hinton (MH) medium for 18–20 h. The bacterial solution was diluted in the same medium for a final inoculum of 5 × 10^3^ colony-forming units (CFU)/mL. Centrifugation and washing removed the medium. The *E. coli* and peptide-treated bacteria samples (after treatment for 30 min) were applied onto mica and imaged in air using the contact mode and recorded with 512 × 512 pixels. The images of HeLa cells and peptide-treated HeLa cells (after treatment for 5 min) were obtained in Dulbecco’s Modified Eagle’s medium (DMEM) using the Acoustic AC (AAC) mode and recorded with 512 × 512 pixels [19]. Alternatively, the adsorbed HeLa cells were fixed with 4% paraformaldehyde for 30 min before imaging with AFM. The morphology of *E. coli* and HeLa cells was modified using Gwyddion software [26]. 

**Ethical Standards:** The authors declare that the experiments of the present study comply with the current laws of China.

## 5. Conclusions

There is a positive correlation between peptide helicity and antimicrobial and anticancer activity. We used a rational approach, with substitutions of d-amino acids on the non-polar and polar faces of the membrane-active peptide V13K, to optimize biological activity by decreasing peptide helicity and hydrophobicity, which is important for the design of MAPs with better therapeutic actions against bacteria and cancer. Based on an AFM study, it is clear that MAP disrupts membrane integrity and exposes the cytoskeleton of bacterial and cancer cells, which provides direct evidence for the mechanism of action of MAPs.

## Figures and Tables

**Table 1:** Design and sequence of α-helical antimicrobial peptides.

Group	Peptide	Amino Acid Sequence ^a^
Parent	V13K	Ac-K-W-K-S-F-L-K-T-F-K-S-A-K-K-T-V-L-H-T-A-L-K-A-I-S-S-amide
Polar face group	K14_D_	Ac-K-W-K-S-F-L-K-T-F-K-S-A-K-*K*-T-V-L-H-T-A-L-K-A-I-S-S-amide
	S11_D_/K14_D_	Ac-K-W-K-S-F-L-K-T-F-K-*S*-A-K-*K*-T-V-L-H-T-A-L-K-A-I-S-S-amide
	K14_D_/T15_D_	Ac-K-W-K-S-F-L-K-T-F-K-S-A-K-*K*-*T*-V-L-H-T-A-L-K-A-I-S-S-amide
	S11_D_/K14_D_/T15_D_	Ac-K-W-K-S-F-L-K-T-F-K-*S*-A-K-*K*-*T*-V-L-H-T-A-L-K-A-I-S-S-amide
Non-polar face group	A12_D_	Ac-K-W-K-S-F-L-K-T-F-K-S-*A*-K-K-T-V-L-H-T-A-L-K-A-I-S-S-amide
	F9_D_/A12_D_	Ac-K-W-K-S-F-L-K-T-*F*-K-S-*A*-K-K-T-V-L-H-T-A-L-K-A-I-S-S-amide
	A12_D_/V16_D_	Ac-K-W-K-S-F-L-K-T-F-K-S-*A*-K-K-T-*V*-L-H-T-A-L-K-A-I-S-S-amide
	F9_D_/A12_D_/V16_D_	Ac-K-W-K-S-F-L-K-T-*F*-K-S-*A*-K-K-T-*V*-L-H-T-A-L-K-A-I-S-S-amide

^a^ Amino acids are represented by one-letter codes; the bold italic letters denote d-amino acid substitutions, all other amino acids are l-amino acids.

**Table 2:** Biophysical data of the peptide analogs.

Peptides ^a^	*t_R_* (min) ^b^	Benign ^c^		50% TFE ^d^	
	25 °C	[θ]_222_	α-Helix (%) ^e^	[θ]_222_	α-Helix (%) ^e^
V13K	44.48	−5617.46	11.1%	−50,520.3	100%
K14_D_	41.93	−2223.13	4.4%	−38,897.2	77.0%
S11_D_/K14_D_	40.77	−3524.85	7.0%	−25,931.3	51.3%
K14_D_/T15_D_	40.41	−1862.4	3.7%	−28,551.5	56.5%
S11_D_/K14_D_/T15_D_	39.59	−2845.85	5.6%	−19,537.5	38.7%
A12_D_	41.46	−4130.9	8.2%	−35,785.1	70.8%
F9_D_/A12_D_	40.04	−2319.89	4.6%	−19,328.6	38.3%
A12_D_/V16_D_	39.79	−3435.72	6.8%	−23,452.9	46.4%
F9_D_/A12_D_/V16_D_	39.06	−1907.83	3.8%	−18,393.1	36.4%

^a^ Peptides are ordered by relative hydrophobicity. ^b^ *t_R_* is the RP-HPLC retention time at 25 °C. ^c^ KP buffer (100 mM KCl, 50 mM KH_2_ PO_4_ /K_2_ HPO_4_, pH 7.4). ^d^ KP buffer with 50% TFE. ^e^ The helical content (%) of the peptides relative to the molar ellipticity value of the parent peptide V13K in 50% TFE.

**Table 3:** MIC and MHC of peptides against bacteria.

Peptides	MHC ^a^ (μM)	G^+^ MIC ^b^ (μM)		G^−^ MIC ^b^ (μM)	
		*S. aureus*	*S. epidermidis*	*P. aeruginosa*	*E. coli*
		ATCC25923	ATCC12228	ATCC27853	ATCC25922
V13K	250	32	4	8	8
K14_D_	125	32	16	16	32
S11_D_/K14_D_	125	64	16	32	64
K14_D_/T15_D_	250	64	16	32	64
S11_D_/K14_D_/T15_D_	250	64	16	125	125
A12_D_	125	64	16	16	32
F9_D_/A12_D_	250	125	64	32	125
A12_D_/V16_D_	>500	125	125	32	125
F9_D_/A12_D_/V16_D_	>500	125	125	125	125

^a^ Hemolytic activity (minimal hemolytic concentration) was determined for human red blood (hRBC) cells after incubation with peptides for 2 h. ^b^ Antimicrobial activity (minimal inhibitory concentration) was determined as the lowest minimum concentration of peptides to inhibit microbial growth.

**Table 4:** IC_50_ of peptides against cancer cell lines.

Peptides	IC_50_ (μM) ^a^			
	HeLa	MIA PaCa-2	HPAC	BxPC-3
V13K	16 ± 0.5	24.8 ± 0.6	7.6 ± 4.0	12.5 ± 1.2
K14_D_	15.6 ± 4.1	17.9 ± 2.3	7.2 ± 5.2	20.0 ± 0.8
S11_D_/K14_D_	30.6 ± 0.5	41.3 ± 2.6	25.5 ± 0.5	22.4 ± 2.3
K14_D_/T15_D_	31.6 ± 2.1	47.1 ± 5.3	22.5 ± 0.5	24.1 ± 5.5
S11_D_/K14_D_/T15_D_	41.1 ± 0.5	56.8 ± 3.3	54.2 ± 1.2	45.5 ± 5.6
A12_D_	28.8 ± 0.8	29.9 ± 4.8	15.5 ± 1.7	45.1 ± 1.0
F9_D_/A12_D_	59.8 ± 0.5	38.8 ± 2.9	42.4 ± 3.0	125.0 ± 3.7
A12_D_/V16_D_	60.1 ± 1.2	46.2 ± 3.0	41.4 ± 1.4	125.0 ± 5.9
F9_D_/A12_D_/V16_D_	125.0 ± 3.4	57.9 ± 2.8	95.6 ± 1.8	125.0 ± 1.8

^a^ Anticancer activity and cell toxicity (IC_50_) represent the concentrations at which cell viability was reduced by 50% compared with untreated cells. The values present mean ± SEM of three independent determinations.

**Table 5:** Tryptophan fluorescence emission maxima and intensity of peptides in HEPES buffer or in the presence of three model membranes.

Peptides	HEPES (nm)	PC/PG			PC/Chol			PC/SM/PE/PS/Chol		
		Wavelength (nm)	Blue Shift (nm)	Intensity	Wavelength (nm)	Blue Shift (nm)	Intensity	Wavelength (nm)	Blue Shift (nm)	Intensity
V13K	353	329	24	459	344	9	486	330	23	582
K14_D_	349	332	17	400	349	0	459	329	20	507
S11_D_/K14_D_	347	332	15	346	345	2	414	331	16	363
K14_D_/T15_D_	349	336	13	348	349	0	412	337	12	346
S11_D_/K14_D_/T15_D_	345	339	6	324	343	2	308	335	10	345
A12_D_	351	328	23	386	345	6	464	327	24	413
F9_D_/A12_D_	350	335	15	339	348	2	456	331	19	338
A12_D_/V16_D_	350	336	14	329	348	2	330	337	13	340
F9_D_/A12_D_/V16_D_	346	335	11	300	346	0	261	337	9	316

## Supplementary Materials

Supplementary materials can be found at www.mdpi.com/1422-0067/19/1/67/s1.

## Abbreviations

MAPs	cationic membrane-active peptides
AFM	atomic force microscopy
KP	potassium phosphate
TFE	trifluoroethanol
RP-HPLC	reversed-phase HPLC
MIC	minimal inhibitory concentration
MHC	minimal hemolytic concentration
Chol	cholesterol
PC	chicken egg phosphatidylcholine
PS	porcine brain phosphatidylserine
PE	linear dichroism
PG	*E. coli* phosphatidylglycerol
SM	porcine brain sphingomyelin
TFA	trifluoroacetic acid
CD	Circular Dichroism
MH	Mueller–Hinton
ONPG	o-nitrophenyl-β-d-galactoside
NPN	1-*N*-phenylnaphthylamine
HEPES	4-(2-hydroxyethyl)-1-piperazineethanesulfonic acid
MTT	3-(4,5-dimethyl-2-thiazolyl)-2,5-diphenyl-2H-tetrazolium bromide
DMSO	dimethyl sulfoxide
AAC	Acoustic AC
