# Supplementary material for: Enantiomeric Effect of d-Amino Acid Substitution on the Mechanism of Action of α-Helical Membrane-Active Peptides
Source: Int J Mol Sci. 2017 Dec 27;19(1):67. doi: 10.3390/ijms19010067 (PMC5796017; doi:10.3390/ijms19010067)
Supplement: Supplementary file 1 [file ijms-19-00067-s001.pdf]

# Enantiomeric effect of D-amino acid substitution on mechanism of action of α-helical membrane-active peptides

**Shiyu Sun [1,2], Guangxu Zhao [1,2], Yibing Huang [1,2,3], Mingjun Cai [4,5], Qiuyan Yan [4,5], Hongda Wang [4,5,*] and Yuxin Chen [1,2,3,*]**

[1] Key Laboratory for Molecular Enzymology and Engineering of the Ministry of Education, Jilin University, Changchun 130012, China.
[2] School of Life Sciences, Jilin University, Changchun 130012, China.
[3] National Engineering Laboratory for AIDS Vaccine, Jilin University, Changchun 130012, China.
[4] State Key Laboratory of Electroanalytical Chemistry, Changchun Institute of Applied Chemistry, Chinese Academy of Sciences, Changchun, Jilin 130022, China.
[5] University of Chinese Academy of Sciences, Beijing 100049, China.

* Author to whom correspondence should be addressed; E-Mail: chen_yuxin@jlu.edu.cn (Y. C.); hdwang@ciac.ac.cn (H. W.); Tel.: +86-431-8515-5220 (Y. C.); +86-431-85262684 (H. W.); +86-431-8515-5200 (Y. C.); Fax: +86-431-85262864 (H. W.).

**Keywords:** membrane-active peptide, helicity, hydrophobicity, atomic force microscopy.

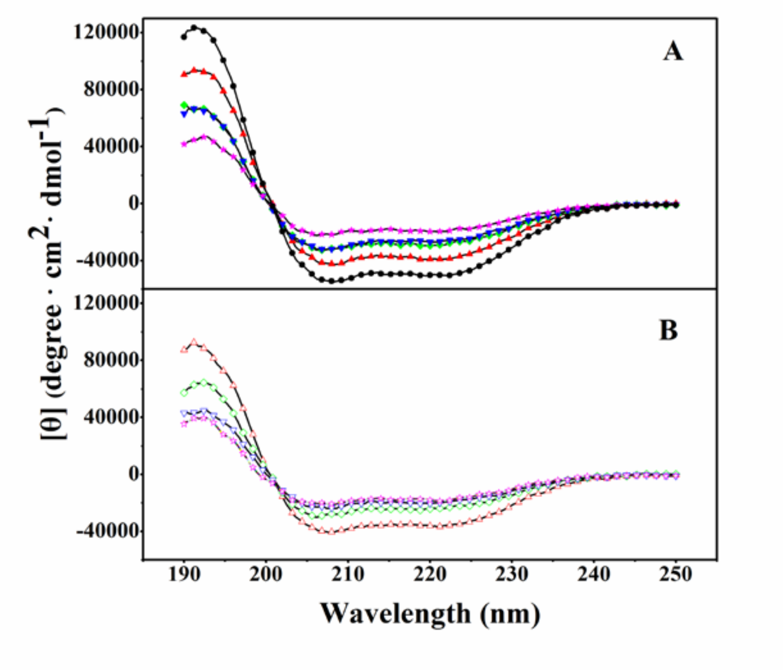


**Figure S1.** CD spectra of peptide analogs. Panels A and B denote the CD spectra of peptides in the KP buffer and 50% TFE, respectively. Symbols used are as follows: ● for V13K; ▲ for K14D; ▼ for S11D/K14D; ◆ for K14D/T15D; ★ for S11D/T14D/T15D; △ for A12D; ▽ for F9D/A12D; ◇ for A12D/V16D; and ☆ for F9D/A12D/V16D.

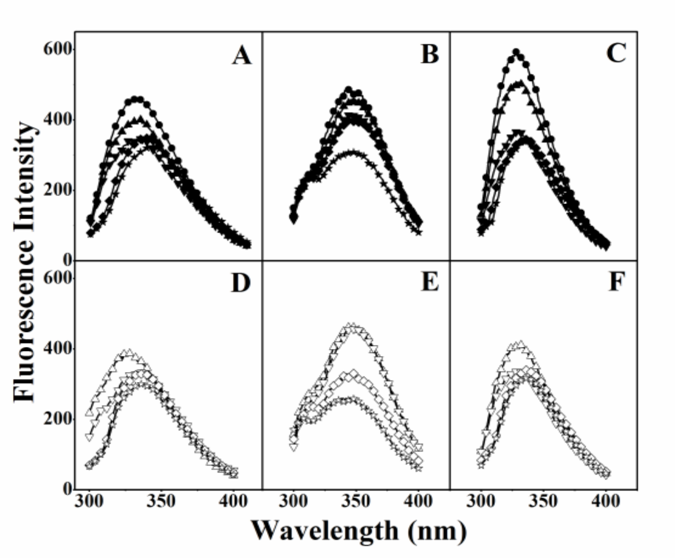


**Figure S2**. Tryptophan fluorescence emission spectra of peptides with three LUV model membranes at 25 °C. Panels A and D show spectra in LUVs mimicking eukaryotic cell membranes (PC/PG (7:3 w/w)); Panels B and E show spectra in LUVs mimicking normal cell membranes (PC/Chol (8:1 w/w)). Panels C and F show spectra in LUVs mimicking cancer cell membranes (PC/SM/PE/PS/Chol (4.35:4.35:1:0.3:1 w/w)) Symbols used are as follows: ● for V13K; ▲ for K14$_{D}$; ▼ for S11$_{D}$/K14$_{D}$; ◆ for K14$_{D}$/T15$_{D}$; ★ for S11$_{D}$/T14$_{D}$/T15$_{D}$; △ for A12$_{D}$; ▽ for F9$_{D}$/A12$_{D}$; ◇ for A12$_{D}$/V16$_{D}$; and ☆ for F9$_{D}$/A12$_{D}$/V16$_{D}$.

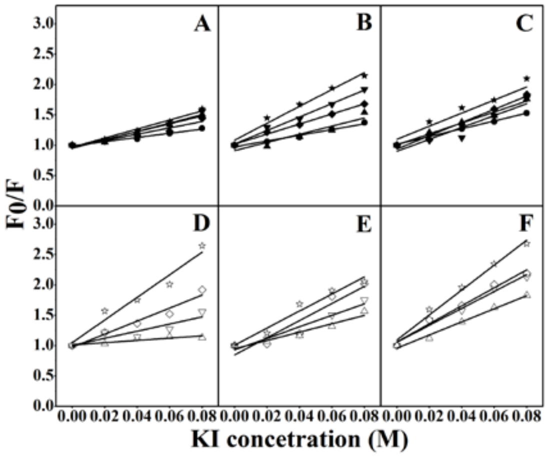


**Figure S3.** Stern–Volmer plots of peptides with three LUV models at 25 °C. Stern–Volmer plots were obtained by the sequential addition of the fluorescence quencher KI. Panels A and D show spectra in LUVs mimicking eukaryotic cell membranes (PC/PG (7:3 w/w)); Panels B and E show spectra in LUVs mimicking normal cell membranes (PC/Chol (8:1 w/w)); Panels C and F show spectra in LUVs mimicking cancer cell membranes (PC/SM/PE/PS/Chol (4.35:4.35:1:0.3:1 w/w)). Symbols used are as follows: ● for V13K; ▲ for K14$_{D}$; ▼ for S11$_{D}$/K14$_{D}$; ◆ for K14$_{D}$/T15$_{D}$; ★ for S11$_{D}$/T14$_{D}$/T15$_{D}$; △ for A12$_{D}$; ▽ for F9$_{D}$/A12$_{D}$; ◇ for A12$_{D}$/V16$_{D}$; and ☆ for F9$_{D}$/A12$_{D}$/V16$_{D}$.
